# Carbon Black Single Piece Electrodes for Nitrate Ion Sensing

**DOI:** 10.3390/molecules30112405

**Published:** 2025-05-30

**Authors:** Martyna Drużyńska, Timo Kikas, Nikola Lenar, Beata Paczosa-Bator

**Affiliations:** 1 Faculty of Materials Science and Ceramics, AGH University of Krakow, Mickiewicza 30, PL-30059 Krakow, Poland; druzynska@agh.edu.pl; 2 Chair of Biosystems Engineering, Institute of Forestry and Engineering, Estonian University of Life Sciences, Kreutzwaldi 56, 51014 Tartu, Estonia; timo.kikas@emu.ee

**Keywords:** carbon black, carbon nanomaterials, single-piece electrode, nitrate sensor, PVC membrane, potentiometry

## Abstract

This work presents an idea for a new reliable potentiometric sensor for nitrate ion determination. The paper provides the optimized procedure for the preparation and maintenance of single-piece nitrate-selective electrodes with carbon black nanoparticles. The optimal carbon black amount was selected based on the electrical properties of the designed sensors. The 5% addition of carbon nanomaterial to the polymeric membrane ensured the highest electrical capacitance (of 610 µF) and the lowest resistance (of 421 kΩ); therefore, this amount was further applied to design single-piece sensors. The influence of carbon black incorporation into the membrane on the analytical performance of the nitrate-selective electrodes was investigated, focusing on parameters such as sensitivity, selectivity, detection limit, and potential stability. Carbon black-modified nitrate-selective sensors exhibit a near-Nernstian response in a wide range of nitrate ion concentrations (with a detection limit of 7 × 10^−7^). Remarkable potential stability, ensured by the hydrophobic properties of the modified membrane, was evaluated during the water-layer test and the calculated potential drift equaled 0.052 mV/h.

## 1. Introduction

The growing need for the precise, reliable, and rapid detection of nitrate ions (NO_3_^−^) in environmental and biological samples has stimulated the development of advanced sensing technologies. Nitrate contamination, primarily resulting from agricultural runoff, industrial waste, and improper waste management, causes significant health and environmental risks including the eutrophication of aquatic systems and methemoglobinemia (“blue baby syndrome”) in infants [1,2]. Therefore, the development of efficient, reliable, and sensitive sensors for nitrate detection is critically important for environmental monitoring and public health protection.

Ion-selective electrodes (ISEs) are a robust tool for ion analysis. Over the years, the significant development of ISEs has been observed [3]. All solid-state electrodes are considered the most promising in terms of their advantages not only in excellent analytical performance but also in simple fabrication [4]. This type of construction relies on an ion-selective membrane, whose basic composition contains an ionophore, lipophilic salt, and polymer matrix [5,6]. Coated-wire electrodes were introduced as the first-ever solid sensors; however, they had many disadvantages, such as potential instability caused by a low double-layer capacitance and a high charge transfer resistance [7,8]. The idea, presented by Cadogan et al. [9] in 1992, of using conducting polymer as a connector between an ionic and electronic conductor pioneered a new type of sensor—solid-contact electrodes. The application of redox-active, hydrophobic material with a high surface area as an internal layer improves the potentiometric response [10]. Although solid-contact ISEs were a great success, the fabrication of this type of sensor requires an additional step: the intermediate solid-contact layer. In 1995, Bobacka et al. presented a simpler method to construct all-solid-state electrodes, called a single-piece electrode (SPE) [11]. The main feature of SPEs is the direct incorporation of the active material into the liquid ion-selective membrane.

A wide range of active materials can be used as ion-to-electron transducers to fabricate single-piece electrodes, such as poly(3-hexylthiophene) (PHT) [12]; polyaniline (PANI) [13]; multi-walled carbon nanotubes (MWCNTs) [14]; 7,7,8,8-tetracyanoquinodimethane (TCNQ) [15]; Molecularly Imprinted Polymers (MIPs) [16]; ionic liquids, e.g., 1-ethyl-3-methylimidazolium chloride (EMImCl) [17]; and monolayer-protected clusters (MPCs) [18]. Introducing new materials directly into the membrane creates an opportunity for the development of single-piece electrodes.

Plasticized poly(vinyl chloride) (PVC) is the most-used matrix for solvent polymeric membranes. PVC ensures the mobility of the membrane and provides sufficient physical properties [19]. Recent advancements have focused on modifying conventional ion-selective membranes with nanomaterials to improve their electrochemical performance. Carbon-based nanomaterials, such as carbon nanotubes, graphene, and carbon black have attracted particular interest due to their high electrical conductivity, large surface area, chemical stability, and ability to promote ion-to-electron transduction [20,21]. Among these, carbon black stands out as an affordable and easily processable material offering a promising strategy for the construction of solid-contact ISEs [22]. The pioneering work on integrating carbon black into ion-selective electrodes (ISEs) was conducted by Paczosa-Bator [23]. This research demonstrated that carbon black could serve effectively either as an intermediate layer between the ion-selective membrane and the electronic conductor or as a component within the membrane itself. The incorporation of carbon black enhanced the electrodes’ potential stability and minimized potential drift.

In this work, we have developed a nitrate-selective polymetric PVC-based membrane with dispersed carbon black nanoparticles to obtain single-piece electrodes without any additional layer. We have optimized the procedure for the electrode’s preparation, using different amounts of carbon black to look into differences between membrane properties. Those membranes were applied onto glassy carbon (GC) disk electrodes to create robust single-piece electrodes for nitrate ion determination. The influence of carbon black’s incorporation into the membrane on the potentiometric performance of the nitrate-selective electrodes was investigated, focusing on parameters such as sensitivity, selectivity, detection limit, and potential stability.

## 2. Results

### 2.1. Membrane Characteristics

#### 2.1.1. Microstructure

**Figure 1:**
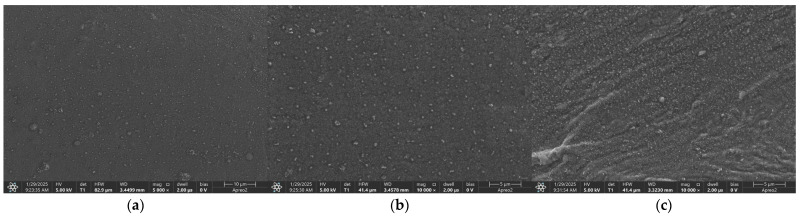
SEM scans of a carbon black-modified nitrate-selective PVC membrane at increasing magnitudes—(**a**) 5000×, (**b**) 10,000×, (**c**) 10,000×.

Scanning Electron Microscopy (SEM) was used to examine the microstructure of a PVC membrane modified with carbon black nanoparticles. For this experiment, a nitrate-selective membrane with 5 mg of carbon black was observed with the electron microscope. The membrane was observed with increasing magnitude from 5000 to 10,000×. Visible spots are carbon black nanoparticles surrounded by PVC. SEM scans, presented in Figure 1, prove that the distribution of carbon black nanoparticles through the membrane is even. This test confirmed that carbon black does not tend to agglomerate in the polymeric membrane and that the proposed preparation method for membrane fabrication is adequate. The performed analysis confirmed the nanometric size of the carbon black particles, which ensures a high surface area, thus resulting in the high electrical capacity of the polymeric membrane.

#### 2.1.2. Wettability

**Figure 2:**
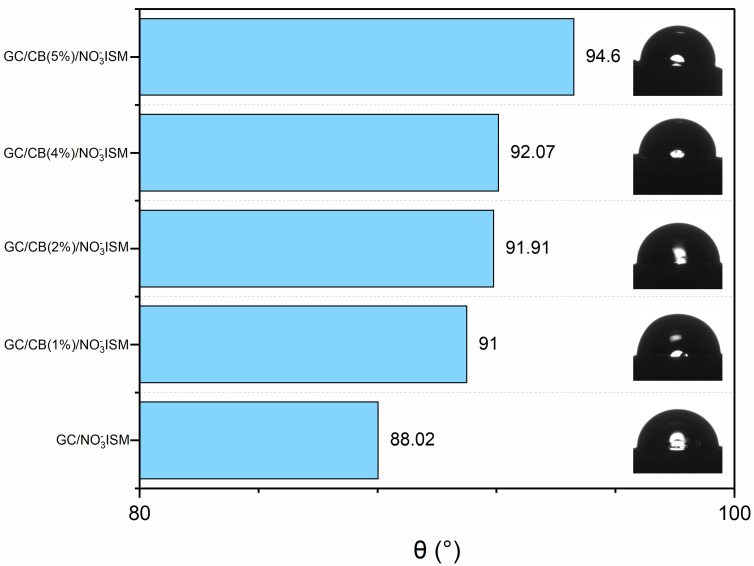
Contact angle values obtained for the designed nitrate-selective PVC membranes with increasing carbon black content.

The wettability test was performed with the use of a tensiometer, which allows for the measurement of the contact angle of materials. A water droplet of 5 µL was discharged from a syringe onto the electrode covered with the nitrate-selective membrane. All designed membranes were tested: a non-modified PVC membrane and four modified membranes with increasing concentrations of carbon black (from 1 to 7 mg of carbon black nanoparticles). The carbon black content in the polymeric membranes was 1 mg (1% (*w/w*%)), 3 mg (2% (*w/w*%)), 5 mg (4% (*w/w*%)), and 7 mg (5% (*w/w*%)). The results are presented in Figure 2. It was observed that with the increased concentration of carbon black, the contact angle also increased. This may be due to the fact that carbon black nanoparticles themselves are characterized by hydrophobic properties. For the non-modified PVC membrane, the contact angle was 88 degrees, while for the carbon black-modified membrane, the contact angle was from 91 to 95 degrees. With ion-selective electrodes, hydrophobic properties of the sensing part of the electrode are desired to ensure a long lifetime of the electrode. Otherwise, water from analyzed solutions may form a thin film underneath the membrane and, as a consequence, lead to the delamination of the polymer from the electrodes’ surface. This phenomenon is explained in detail in the Section 3.

#### 2.1.3. Electrical Properties

The electrical properties of the designed membranes were tested using chronopotentiometry and electrochemical impedance spectroscopy. All electrochemical techniques were applied to determine electrical capacitance and resistance parameters. The electrical parameters of the modified membranes were compared with the properties determined for the non-modified nitrate-selective membrane.

##### Chronopotentiometry

**Figure 3:**
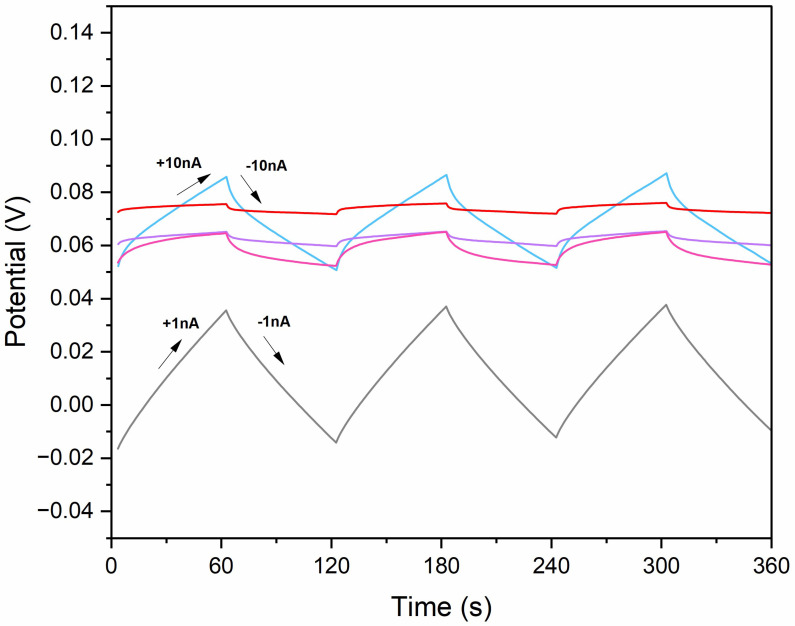
Chronopotentiograms recorded for GC/CB(1%)/NO_3_-ISM (blue line), GC/CB(2%)/NO_3_-ISM (purple line), GC/CB(4%)/NO_3_-ISM (pink line), GC/CB(5%)/NO_3_-ISM (red line), and GC/NO_3_-ISM (grey line) with the current flow of 1 nA or 10 nA in 10^−1^ M KNO_3_ solution.

In this technique, a current of positive and negative signs, alternately, was passed through the three-electrode cell with 10^−1^ M potassium nitrate as an electrolyte. The value of the current was set at 1 nA (for coated-disc electrodes) or 10 nA (for single-piece electrodes) and the achieved potential–time graph is presented in Figure 3.

According to the obtained chronopotentiogram, the potential drift of the modified electrodes given by the dE/dt ratio was 6 × 10^−4^ V/s, 7 × 10^−4^ V/s, 0.014 V/s, and 6 × 10^−5^ V/s for GC/CB(1%)/NO_3_-ISM, GC/CB(2%)/NO_3_-ISM, GC/CB(4%)/NO_3_-ISM, GC/CB(5%)/NO_3_-ISM, and GC/NO_3_-ISM electrodes, respectively. Based on the potential drift values and the equation proposed by Bobacka in 1999 [24], the electrical capacitance was calculated for the linear part of the slope and equaled 23, 227, 302, and 610 µF for the GC/CB(1%)/NO_3_-ISM, GC/CB(2%)/NO_3_-ISM, GC/CB(4%)/NO_3_-ISM, and GC/CB(5%)/NO_3_-ISM sensors, respectively. In comparison, the electrical capacitance of the non-modified membrane was 1.22 µF. The test proved that the modification of a nitrate-selective membrane with carbon black nanoparticles allows for the enhancement of the electrical capacity of the sensor. The highest electrical capacitance parameter is attributed to the membrane with 7 mg (5%) of carbon black.

The exact same experiment was used for the evaluation of the resistance parameter. With the use of the equation in [24], where dE_dc_ stands for the potential jump between two steps and I is the current applied during the experiment. The resistance of the charge transfer in carbon black-based electrodes was 655, 549, 450, and 421 kΩ for GC/CB(1%)/NO_3_-ISM, GC/CB(2%)/NO_3_-ISM, GC/CB(4%)/NO_3_-ISM, and GC/CB(5%)/NO_3_-ISM, respectively. For non-modified electrodes, the resistance was 810 kΩ. The addition of 7 mg (5%) of carbon black to the membrane allows for a reduction in resistance. All calculated parameters are summarized in Table 1.

(1) $$R=\frac{I}{dE_{dc}}$$

According to the results obtained from chronopotentiometry and wettability measurements, the electrode with the best performance was chosen. Sensor GC/CB(5%)/NO_3_-ISM with the addition of 7 mg of carbon black is characterized by the highest electrical capacity and contact angle value; therefore, the following measurements will be presented for this electrode.

##### Electrochemical Impedance Spectroscopy

**Figure 4:**
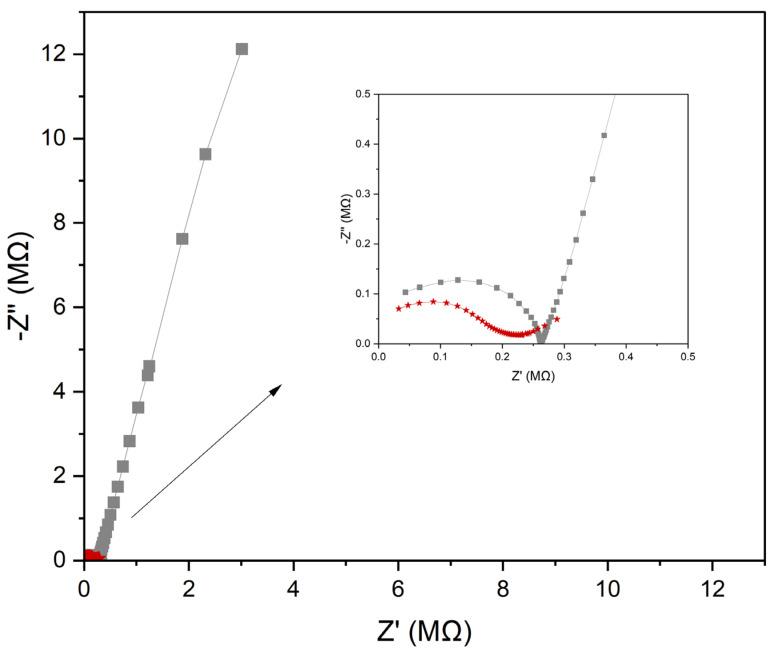
Nyquist plots recorded in 10^−1^ M standard KNO_3_ solution for GC/CB(5%)/NO_3_-ISM (red line) and GC/NO_3_-ISM (grey line). The inset presents the enlarged part of the plot.

The other technique used for electrode characterization is electrochemical impedance spectroscopy. With this technique, the most common way to present results is the Nyquist plot on which the imaginary part of the impedance (Z”) is plotted as a function of the real impedance (Z’). The Nyquist plots for all studied electrodes are presented in Figure 4. The capacitance value can be calculated for low frequencies (in this case f = 0.01 Hz) and equals 1.31 and 398 μF for the GC/CB(5%)/NO_3_-ISM, and GC/NO_3_-ISM electrodes, respectively.

The resistance value can be obtained from the Nyquist plot as a diameter of a semicircle and equals 270 kΩ for the GC/NO_3_-ISM electrode and 155 kΩ for the GC/CB(5%)/NO_3_-ISM electrode. Similarly, as in the case of the chronopotentiometry method, this measurement shows that the addition of carbon black to the membrane allows for an increase in electrical capacitance and a decrease in the resistance parameter.

### 2.2. Analytical Properties of Sensor

After performing the membrane characterization, the electrode with 5% carbon black (GC/CB(5%)/NO_3_-ISM) was decided to have the most favorable parameters. Hence, in the following part of the paper, the analytical properties are presented for the (GC/CB(5%)/NO_3_-ISM) electrode. The coated-disc (GC/NO_3_-ISM) sensor was used as a control electrode.

#### 2.2.1. Ionic Response

**Figure 5:**
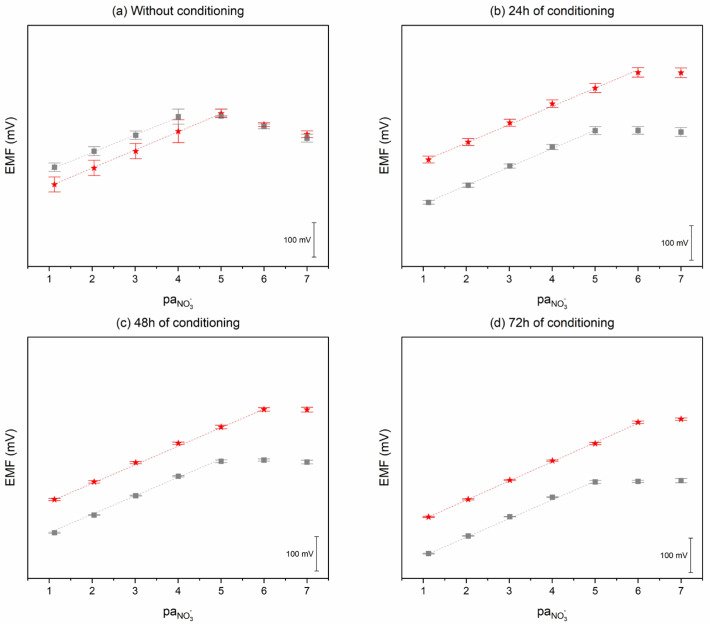
Potentiometric response without and after 24, 48, and 72 h of conditioning in 10^−5^ M KNO_3_ standard solution of GC/CB(5%)/NO_3_-ISM (red star) and GC/NO_3_-ISM (grey square) electrodes towards nitrate ions examined in KNO_3_ solutions.

The sensors’ response towards nitrate ions was evaluated based on the parameters of calibration curves. Three calibrations were performed on consecutive days for carbon black-based and coated-wire electrodes. Calibrations were conducted after 24, 48, and 72 h from the beginning of electrodes’ conditioning in 10^−5^ M standard KNO_3_ solution. During the measurements, electrodes were continuously stored in the conditioning solution. The potentiometric response (the relationship between EMF and pNO_3_^−^ value) of the designed sensors is presented in Figure 5. The indicating factor of reproducibility was the standard deviation value. It can be seen that with the increase in the conditioning period, the standard deviation values, presented as error bars, decrease. It may be concluded that the designed sensors require at least 72 h of conditioning in 10^−5^ M KNO_3_ solution to reach satisfactory repeatability. The pH of the KNO_3_ calibration solutions was 5.5 ± 0.1.

Analytical parameters, calculated based on the results obtained for each group, are presented in Table 2. It can be seen that the reproducibility of the designed sensors, given by the standard deviation values calculated based on the results obtained within the group, was improved due to the addition of carbon black to the membrane.

**Figure 6:**
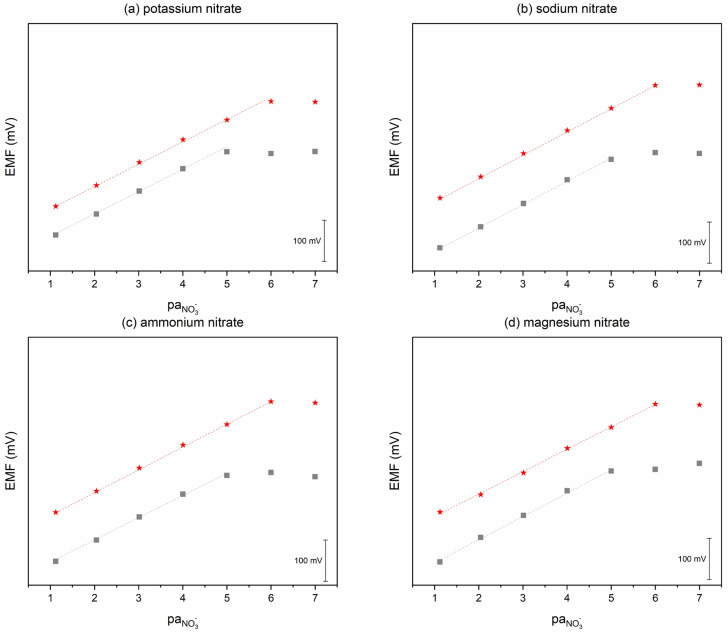
Exemplary potentiometric response of GC/CB(5%)/NO_3_-ISM (red star) and GC/NO_3_-ISM (grey square) electrodes towards nitrate ions examined in different nitrate salts: (**a**) potassium nitrate, (**b**) sodium nitrate, (**c**) ammonium nitrate, (**d**) magnesium nitrate.

The effect of the ionic response towards nitrate ions in the presence of various cations in the solution on the potentiometric response was examined. The response was tested using different nitrate salts: potassium, sodium, ammonium, and magnesium ions. No significant influence of the cation on the potentiometric response was observed. The results are presented in Figure 6.

#### 2.2.2. Water Layer Test

The possibility of the formation of a water layer was evaluated during the water layer test. The water layer test was conducted according to the procedure presented by Fibbioli et al. in [8] and Guzinski et al. in [25]. The designed GC/CB(5%)/NO_3_-ISM electrodes were placed into 10^−2^ M NO_3_^−^ ions standard solution for 5 h, then into 10^−2^ M Cl^−^ ions standard solution for several hours, and eventually placed back into the nitrate ion solution for a longer period of time. During the test, the EMF was recorded in time to observe the possible potential drift when changing the primary ion in the analyzed solution.

**Figure 7:**
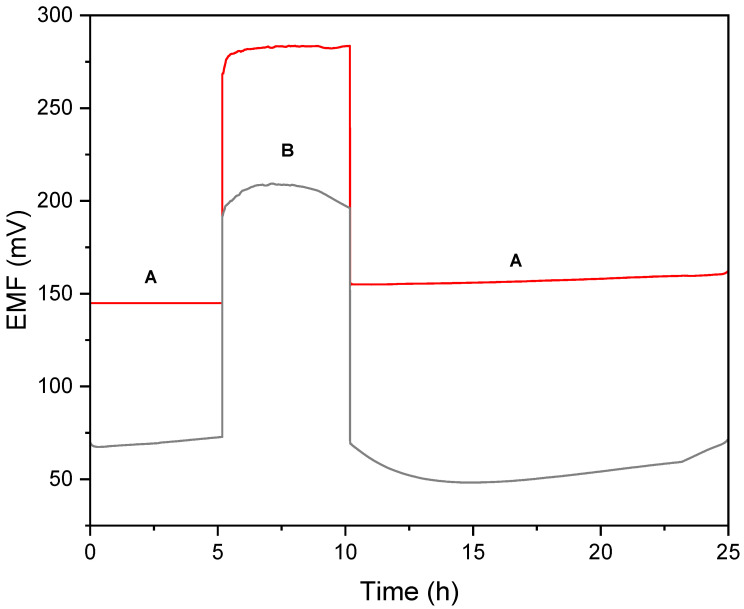
Water test performed in 10^−2^ M standard KNO_3_ (A) and 10^−2^ M standard KCl (B) solutions for GC/CB(5%)/NO_3_-ISM (red line) and GC/NO_3_-ISM (grey line) sensors.

As presented in Figure 7, for the GC/CB(5%)/NO_3_-ISM electrode, the potential drift was not observed when changing the KNO_3_ into the KCl solution, and the potentiometric response was immediate and stable. In contrast, the electrode without the addition of carbon black exhibited a substantial drift of EMF, indicating the presence of a water layer formed under the ion-selective membrane. The water test confirmed that hydrophobic materials introduced into the construction of ion-selective electrodes prevent the formation of a water layer under the polymeric membrane. The addition of carbon black eliminated the water film, which translated into enhanced potential stability.

#### 2.2.3. Potential Stability

**Figure 8:**
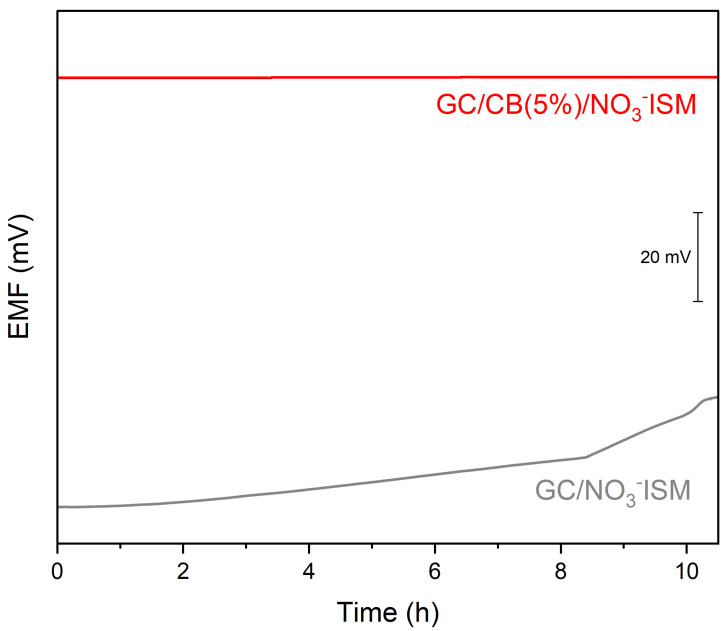
Potential stability test performed in 10^−2^ M standard KNO_3_ solution for the GC/CB(5%)/NO_3_-ISM (red line) and GC/NO_3_-ISM (grey line) sensors.

The potential stability of the designed carbon black-modified sensors was evaluated during the long-time potentiometric measurement in the presence of the non-modified nitrate sensor (used for the comparison) versus the reference electrode. The measurement was conducted in 10^−2^ M nitrate ion solution and lasted 10 h. The potential stability of the sensor is characterized by the potential drift parameter. The calculated potential drift parameters were as follows: 0.052 mV/h for the GC/CB(5%)/NO_3_-ISM sensor and 2.18 mV/h for the GC/NO_3_-ISM sensor. The decrease in the potential drift value is due to the higher electrical capacitance and enhanced hydrophobicity of the single-piece electrode’s membrane. The results are presented in Figure 8.

#### 2.2.4. pH Sensitivity

**Figure 9:**
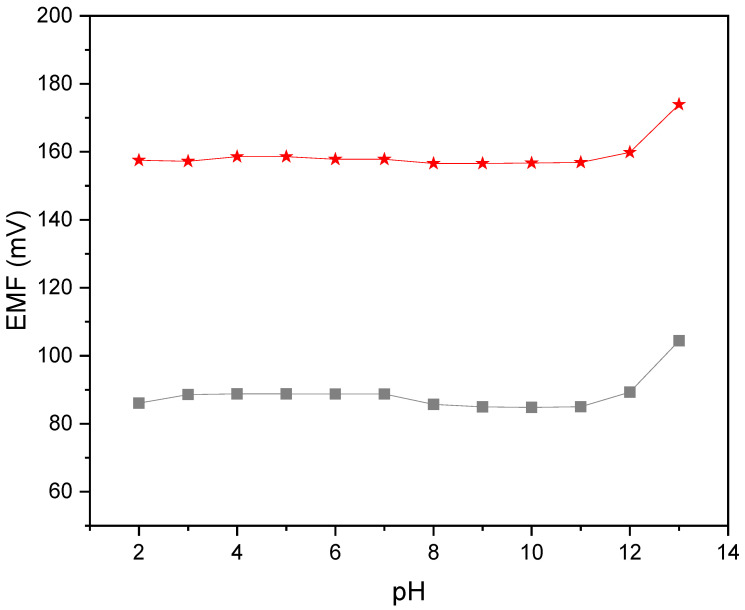
pH test performed for GC/CB(5%)/NO_3_-ISM (red star) and GC/NO_3_-ISM (grey square) electrodes.

A pH sensitivity test was conducted to validate the scope of pH values at which the designed sensors can be applied. The test was conducted in the solutions of a constant nitrate ion concentration and changing pH values (from 2 to 13). Standard nitrate ion solution was titrated with 3 M sodium hydroxide solution to obtain solutions of pH from 6 to 13, and 3 M hydrochloric acid solution was used to obtain lower pH values. As can be seen in Figure 9, stable EMF values were observed for solutions of pH from 2 to 11. The test showed that electrodes with carbon black-modified membranes exhibit stable potentiometric response in a wide range of pH values between 2 and 11. The process was controlled using the glass electrode.

#### 2.2.5. Redox Sensitivity

**Figure 10:**
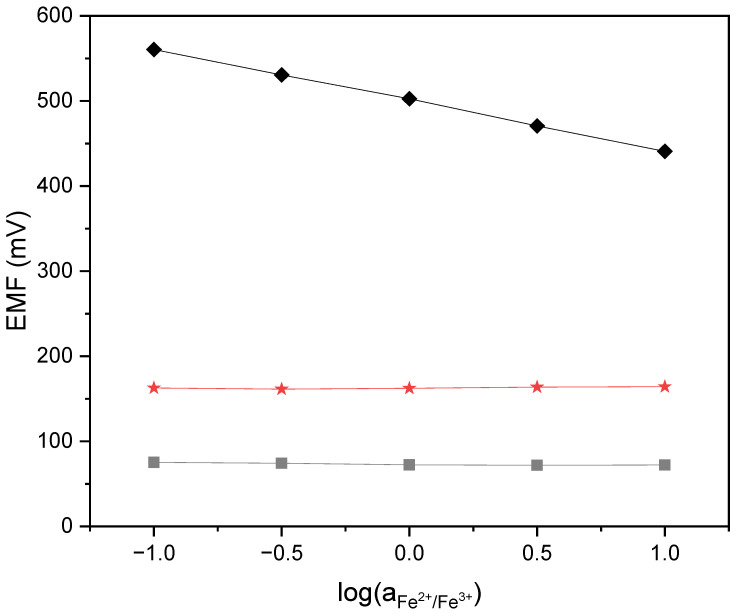
Redox test performed for GC/CB(5%)/NO_3_-ISM (red star) and GC/NO_3_-ISM (grey square) electrodes in the presence of a glassy carbon disc electrode (black diamond) in solutions of varying activity of an FeCl_2_/FeCl_3_ redox couple.

Carbon black as a carbon nanomaterial exhibits redox sensitivity. The presence of redox couples in the analyzed solutions may affect the potentiometric response if the electrode is sensitive to changing redox conditions. This phenomenon is not desirable. To assess whether the designed sensors exhibit a potentiometric response towards redox couples, the experiment involving an FeCl_2_/FeCl_3_ redox couple of changing concentrations was conducted, as presented in Figure 10. A glassy carbon disc electrode was used as a control electrode.

The glassy carbon disc electrode without an ion-selective membrane exhibits a quasi-Nernstian response towards the changing activity of the redox couple, while no response was observed for both GC/CB(5%)/NO_3_-ISM and GC/NO_3_-ISM electrodes. The test shows that despite the presence of redox-sensitive carbon black in the polymeric membrane, the membrane surrounding the carbon particles acts as an insulator for carbon black, and thus, the designed sensors did not exhibit any redox sensitivity.

### 2.3. Application of Sensors

The designed sensors find their application in environmental monitoring. Monitoring nitrate levels in surface waters is very important to prevent eutrophication [26]. Increasing concentrations of nitrates in water bodies can lead to a reduction in oxygen, which results in disturbances in the ecosystem.

To demonstrate the feasibility of analyses with the designed electrodes, the analysis of nitrate concentrations in surface waters was conducted. The samples were collected from the rivers Wisłok (Rzeszów, Poland), Wisła, and Rudawa (Kraków, Poland) and the lake Zakrzówek (Kraków, Poland). In the first step, the collected water samples were filtrated. In the following step, 10 mL of supporting electrolyte CH_3_COONa and 90 mL of filtrate were mixed. The analysis was performed using the calibration curve method. The results of the analysis are presented in Table 3.

The sensors presented in this work can be successfully used in the quality control of surface waters. The presented analytical procedure is simple and takes a relatively short time, which can be pointed out as a significant advantage. The repeatability of single-piece sensors, represented by standard deviation, is satisfactory. The recoveries calculated after the addition of standard NO_3_^−^ solution approached 100%, indicating high analytical accuracy.

## 3. Discussion

The obtained results are compared and discussed in this section. Table 4 presents the comparison of parameters of different single-piece electrodes presented so far in the literature with the parameters of the GC/CB/NO_3_-ISM electrode presented in this work.

The designed single-piece carbon black-modified nitrate-selective sensor is characterized by a near-Nernstian response in a wide range of 10^−6^ to 10^−1^ M of nitrate ions and can be considered competitive with the other single-piece electrodes presented so far in the literature. With potentiometric sensors, high electrical capacity and low resistance values are desirable. High electrical capacity is the most desirable feature of ISEs. The ability of the sensor to store the charge, determined by the double layer/redox capacitance parameter, enables it to sustain the equilibrium in the presence of external disturbances and protects the electrode from the impact of the current flow during the potentiometric measurement. Electrodes characterized by a high electrical capacitance parameter exhibit stable potentiometric response in time and insensitivity to the perturbations that may occur during the measurement, e.g., power fluctuations. The designed nitrate-selective sensor exhibits an electrical capacitance that equals 610 µF. In comparison, electrodes modified conducting polymer-PANI exhibit a capacitance of only 15 µF [16]. Great electrical capacity can be attributed to the single-piece electrodes with the addition of carbon nanomaterials. Liu et al. presented Pb-selective electrodes with carbon nanotubes and obtained an electrical capacitance of 50 µF [14]. Paczosa-Bator was the first to introduce carbon-black to potentiometric sensors [23], and in her 2012 work, single-piece electrodes with a capacitance of 429 µF were designed. In our previous work, we presented single-piece electrodes with graphene, carbon nanotubes, and carbon black [27]. Thanks to the optimization procedure, we were able to elevate the electrical capacitance value from 211 to 610 µF and consequently decrease the potential drift from 0.087 to 0.052 mV/h. In comparison with other materials, carbon black allows us to obtain a potentiometric sensor with great potentiometric response stability. Paczosa-Bator was able to obtain a single-piece electrode with a potential drift of only 0.009 mV/h. This may be due to the fact that carbon black itself is characterized by hydrophobic properties. With ion-selective electrodes, hydrophobic properties are desired, as they prevent the formation of a water layer underneath the membrane. Ion selective electrodes, after being transformed from conventional electrodes with an internal solution into all-solid-state electrodes with a membrane placed directly onto the electronic conductor, were characterized by the issue of water layer formation. After being repeatedly placed into water solutions during a series of measurements and conditioning, all-solid-state electrodes tend to absorb water, and a water layer is formed at the interface between the ion-selective membrane and the electrode’s surface. The presence of this thin water film leads to the potential drift of potentiometric response and may be the cause of shortening the lifetime of electrodes by deteriorating the adherence of the membrane to the electrode.

Contact angle measurements established that the obtained carbon-black modified membrane is characterized by hydrophobic properties and the water test conducted during the potentiometric measurement proved that the obtained electrodes are free of a water layer. This phenomenon explains the remarkable potential stability of all carbon black single-piece sensors.

The small value of resistance can be attributed to the facilitated charge transfer processes between electronic and ionic conductors. The designed GC/CB/NO_3_^−^ sensor is characterized by a resistance parameter of only 421 kΩ.

## 4. Materials and Methods

### 4.1. Sensor Preparation

The procedure of single-piece electrode preparation is based on introducing a modifier (carbon black) directly into an ion-selective membrane. The preparation process is simpler and takes less time compared to the preparation of solid-contact electrodes with separate layers. The first step is the preparation of the GC disk electrodes’ surface by polishing them in alumina slurries and rinsing them with water and methanol.

The components of the PVC-based membrane contain (*w/w*%) polyvinyl chloride (35%) plasticizer (65%), ionophore (1.1%), and lipophilic salt (0.7%). These components were dissolved in tetrahydrofuran, then divided into five separate vials. In each vial with a nitrate-selective membrane, carbon black was added in different amounts: 1 mg (1% (*w/w*%)), 3 mg (2% (*w/w*%)), 5 mg (4% (*w/w*%)), and 7 mg (5% (*w/w*%)). One vial was left without additives so that a control electrode could be prepared.

Membranes were cast onto a surface of GC electrodes using a drop-casting technique, with a volume of 70 µL. Three items of electrodes, representing each group, were prepared: GC/CB(1%)/NO_3_-ISM, GC/CB(2%)/NO_3_-ISM, GC/CB(4%)/NO_3_-ISM, and GC/CB(5%)/NO_3_-ISM.

**Figure 11:**
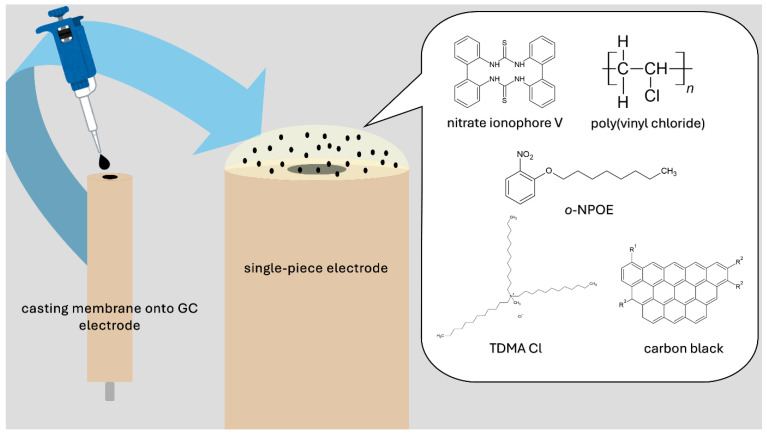
Schematic representation of the designed carbon black-based ion-selective electrode.

The whole procedure took only a few minutes and required minimal use of chemicals. It was performed using only basic laboratory equipment. The schematic representation of designed potentiometric sensors is presented in the Figure 11.

### 4.2. Chemicals

The membrane components included polyvinyl chloride (PVC) of high molecular weight, lipophilic salt tridodecylmethylammonium chloride (TDMA Cl), plasticizer 2-nitrophenyl octyl ether (o-NPOE), and nitrate ionophore V. All components were dissolved in tetrahydrofuran (THF). All membrane components and a solvent were purchased from Sigma Aldrich, St. Louis, MO, USA. Carbon black (CB), used as a membrane modifier, was obtained from 3D-nano, Krakow, Poland.

As a standard nitrate ion solution, potassium nitrate aqueous solutions were used. Potassium nitrate was purchased from POCH (Gliwice, Poland). The standard NO_3_^−^ ion solutions were used for all the conducted measurements, including potentiometry, electrochemical impedance spectroscopy, and chronopotentiometry. For the additional examination and validation of the designed sensors, ferrous(II) chloride, ferric(III) chloride, potassium chloride, magnesium nitrate, ammonium nitrate, and sodium nitrate were used (all purchased from POCH).

### 4.3. Methods

The main electrochemical technique used for the examination and validation of the designed sensors was potentiometry. Experiments conducted to determine the analytical parameters were performed using a 16-channel mV-meter (Lawson Labs, Inc., Malvern, PA, USA). The potentiometric response towards nitrate ions was examined in standard KNO_3_ solutions of 10^−7^ to 10^−1^ M concentration. Potentiometric measurements were conducted versus the reference electrode—Ag/AgCl electrode with a 3 M KCl solution (6.0733.100 Metrohm, Herisau, Switzerland) and in the presence of an auxiliary electrode (platinum wire).

The chronopotentiometry, cyclic voltammetry, and electrochemical impedance spectroscopy measurements were carried out with the use of an Autolab General Purpose Electrochemical System (AUT302N.FRA2-AUTOLAB, Metrohm Autolab, Barendrecht, The Netherlands) with NOVA 2.1. software. Designed single-piece ISEs were tested as working electrodes in a three-electrode cell with a Ag/AgCl reference electrode with a 3 M KCl solution (6.0733.100 Metrohm, Herisau, Switzerland) and in the presence of an auxiliary electrode (glassy carbon electrode). Chronopotentiometric tests were conducted in the 10^−1^ M standard KNO_3_ solution. A constant current of +1 nA was applied to the working electrode for 60 s, followed by a −1 nA current for another 60 s [24]. For carbon black-modified electrodes, a current of 10 nA was applied.

Nyquist plots were recorded during the measurement of electrochemical spectroscopy impedance, performed in the 10^−1^ M standard KNO_3_ solution. The impedance spectra were obtained by applying a frequency from 100 kHz to 0.01 Hz using an AC amplitude of 10 mV superimposed on 0.15 V versus the reference electrode.

The microstructure of nitrate-selective membranes was examined using a scanning electron microscope. SEM scans were collected using Scanning Electron Microscope-Apreo 2 (Thermo Scientific, Waltham, MA, USA).

The wettability measurements were carried out using a Theta Lite microscope by Biolin Scientific (Gothenburg, Sweden). A water droplet of 5 µL was discharged onto the membranes’ surface. The contact angle was measured using One Attension 4.0 software.

The pH test was conducted by the titration of a 0.01 M KNO_3_ solution with 3M HCl and NaOH. The titration process was controlled using the glass electrode (6.0150.100 Metrohm, Switzerland).

## 5. Conclusions

In this study, novel nitrate-selective electrodes, based on poly(vinyl chloride) (PVC) membranes modified with carbon black, were successfully developed and evaluated. The incorporation of carbon black as an additive to the membrane significantly enhanced the electrodes’ performance by improving ion-to-electron transduction, leading to superior potential stability, lower detection limits, and faster response times compared to coated-disc electrodes. By incorporating carbon black directly into the membrane, it was possible to avoid introducing an additional material layer into the electrode, which simplified the fabrication procedure and reduced the preparation time. The paper provides the optimization procedure and, according to the results from wettability and electrochemical tests, the optimal carbon black content in polymeric membranes is 5%. This content ensured the highest contact angle of the membrane and the highest electrical capacity, which translated into great analytical properties of the electrodes, such as remarkable potential stability.

Given its low cost, high conductivity, and ease of incorporation, carbon black represents an attractive material for advancing the design of next-generation ion-selective electrodes. The proposed nitrate sensor offers a promising solution for environmental monitoring and could be adapted for the detection of other ions through appropriate membrane components (mainly ionophores). As a practical application of single-piece nitrate-selective electrodes, we conducted nitrate ion analysis in river waters, receiving great repeatability.

Future work will focus on the further optimization of membrane content, the miniaturization of the sensor platforms, and the validation of their performance in real sample matrices.

## Figures and Tables

**Table 1:** Electrical capacitance parameter values of nitrate-selective electrodes modified with carbon black, obtained using a chronopotentiometry technique.

Electrode Symbol	Current [nA]	Resistance ± SD [kΩ]	Electrical Capacitance ± SD [µF]
GC/NO_3_-ISM	1	810 ± 12	1.22 ± 0.05
GC/CB(1%)/NO_3_-ISM	10	655 ± 10	23.2 ± 0.7
GC/CB(2%)/NO_3_-ISM	10	549 ± 9	227 ± 2
GC/CB(4%)/NO_3_-ISM	10	450 ± 8	302 ± 3
GC/CB(5%)/NO_3_-ISM	10	421 ± 8	610 ± 5

**Table 2:** Analytical parameters of the designed carbon black-based nitrate-selective electrodes and non-modified sensors compared within each group, together with standard deviation values (n = 3 items).

Electrode Symbol	Sensitivity S ± SD [mV]	Standard Potential E^0^ ± SD [mV]	Linear Range [M]	Limit of Detection [M]	Potential Stability [mV/h]
GC/CB(5%)/NO_3_-ISM	56.15 ± 0.50	115.1 ± 0.5	10^−1^–10^−6^	7 × 10^−7^	0.052
GC/NO_3_-ISM	56.60 ± 0.78	7.9 ± 2.2	10^−1^–10^−5^	1 × 10^−5^	2.18

**Table 3:** Results of the determination of nitrate ions in water samples using single-piece electrodes modified with carbon-black (n = 3 samples).

Water Body	Nitrate Concentration ± SD [µg/L]	Recovery [%]	pH
Wisłok	214 ± 1	102	7.6
Wisła	443 ± 7	99	7.7
Rudawa	358 ± 12	98	7.5
Zakrzówek	143 ± 7	101	8.0

**Table 4:** The comparison of the parameters of the single-piece ion-selective electrodes presented so far in the literature.

Additive to Membrane	Ion	Slope [mV/dec]	Linear Range [M]	Electrical Capacity [µF]	Potential Drift	Reference
POT	Li^+^	56.80	10^−1^–10^−3^	-	0.8 mV/day	[11]
PANI	Ca^2+^	28.20	10^−1^–10^−4^	-	0.2 mV/day	
MWCNTs	Na^+^	58.00	10^−1^–10^−6^	-	0.3 mV/day	[19]
	K^+^	59.10	10^−1^–10^−6^	-	0.5 mV/day	
	Cu^2+^	28.90	10^−1^–10^−6^	-	0.3 mV/day	
	Ca^2+^	29.20	10^−1^–10^−6^	-	0.3 mV/day	
1-ethyl-3-methyl imidazolium chloride	Co^2+^	31.80	10^−1^–10^−7^	-	0.15 mV/day	[17]
PANI	2,4-dichlorophenol (DCP)		0.47–13 µM	15	-	[16]
MWCNTs	Pb^2+^	29.00	2 × 10^−3^–2 × 10^−9^	50	-	[14]
CB	K^+^	58.80	10^−1^–10^−6^	429	0.009 mV/h	[23]
CB	NO_3_^−^	54.22	10^−1^–10^−6^	211	0.087 mV/h	[27]
CNTs		54.15	10^−1^–10^−6^	88	0.082 mV/h	
GR		54.32	10^−1^–10^−6^	931	0.065 mV/h	
CB	NO_3_^−^	56.15	10^−1^–10^−6^	610	0.052 mV/h	This work

## Data Availability

Data are contained within the article.
